# Role of lipocalin-2 in surgery-induced cognitive decline in mice: a signal from neuron to microglia

**DOI:** 10.1186/s12974-022-02455-5

**Published:** 2022-04-12

**Authors:** Xuwu Xiang, Xiaodong Tang, Yang Yu, Shulan Xie, Lu Liu, ManLi Chen, Rong Zhang, Xianhui Kang, Yueying Zheng, Guang Yang, Shuyuan Gan, Shengmei Zhu

**Affiliations:** 1 Department of Anesthesiology, The First Affiliated Hospital, Zhejiang University School of Medicine, #79 Qingchun Road, Hangzhou, Zhejiang 310003 People’s Republic of China; 2 2Department of Anesthesiology, Columbia University Medical Center, New York, NY 10032 USA

**Keywords:** Cognitive decline, Surgery, Lipocalin-2, Neuroinflammation, Microglia

## Abstract

**Background:**

Perioperative neurocognitive disorders (PNDs) are common complications observed among surgical patients. Accumulating evidence suggests that neuroinflammation is one of the major contributors to the development of PNDs, but the underlying mechanisms remain unclear.

**Methods:**

qPCR and ELISA analysis were used for detecting LCN2 and cytokine levels. *cx3cr1*^CreER/−^:: *R26*^iDTR/−^ crossed mouse line was used for microglia depletion; intracranial injection of recombinant LCN2 (rLCN2) and adeno-associated viruses (AAV)-mediated shRNA silencing approaches were used for gain and loss of function, respectively. Combing with in vitro microglia cell culture, we have studied the role of LCN2 in surgery-induced cognitive decline in mice.

**Results:**

We revealed that *Lcn2* mRNA and protein levels were greatly increased in mouse hippocampal neurons after surgery. This surgery-induced elevation of LCN2 was independent of the presence of microglia. Gain of function by intracranial injection of rLCN2 protein into hippocampus disrupted fear memory in naive mice without surgery. Conversely, silencing LCN2 in hippocampus by AAV-shRNA protected mice from surgery-induced microglia morphological changes, neuroinflammation and cognitive decline. In vitro, application of rLCN2 protein induced the expression of several pro-inflammatory cytokines in both BV-2 and primary microglia culture.

**Conclusions:**

These data suggest LCN2 acts as a signal from neuron to induce proinflammatory microglia, which contributes to surgery-induced neuroinflammation and cognitive decline in mice.

**Supplementary Information:**

The online version contains supplementary material available at 10.1186/s12974-022-02455-5.

## Background

Memory, attention and other cognitive impairments following major surgical procedures are common complications experienced by surgical patients, which are now termed as perioperative neurocognitive disorders (PNDs) [1–3]. The incidence of PNDs is reported up to around 5.7%- 54.3% in surgical patients [4, 5]. People who suffer from PNDs are at risk of poor surgical outcomes such as increased mortality [6] and decreased quality of life [7]. Moreover, PNDs also have a synergistic relationship with neurodegenerative conditions before surgery [8–10], making their impact even more concerning.

Surgical procedures are associated with robust innate immune activation [11], which could lead to neuroinflammation [12, 13]. Accumulating clinical and preclinical evidence suggest that neuroinflammation is one of the major contributors to the development of PNDs [12, 14–16]. In surgical patients, the increase of inflammatory cytokines in cerebral spinal fluid (CSF) has been repeatedly described [17–20]. In addition, rodent studies reveal that surgical trauma-released alarmins could activate the innate immune system, including the activation of immune cells and the increased production of proinflammatory cytokines [21]; these peripheral immune-related events lead to the disruption of the blood–brain barrier and subsequent recruitment of inflammatory monocytes into brain [22], these together with the subsequently activated microglia [23, 24], disrupt cognitive functions [22, 25].

Lipocalin-2 (LCN2, 26kD) is a small secreted protein [26] and initially described as an anti-bacterial protein for clearing the pathogen [27]. In normal adult brain, the expression of LCN2 is very low and becomes upregulated during injury or neurodegenerative conditions [28]. Our previous genome-wide RNA-seq analysis showed that *Lcn2* was one of the top upregulated genes in mouse hippocampus after surgery [29]. A prior study found an association between elevation of LCN2 levels and cognitive dysfunction after surgery in rats [30]. Consistently, elevation of LCN2 levels have also been observed in plasma and CSF of surgical patients [31]. Several clinical studies have assessed the potential value of LCN2 as a biomarker for neurodegenerations including vascular dementia [32], mild cognitive impairment [33] or preclinical stage of Alzheimer’s disease [34]. However, in the context of sterile surgery evoked conditions, the precise role of LCN2 is unclear. Therefore, we sought to address the role of LCN2 in surgery-induced cognitive decline.

## Materials and methods

### Animals

Twelve- to sixteen-week-old C57BL/6J male mice, and *cx3cr1*^CreER^ (JAX stock: 021160) and *Rosa26*^iDTR^ (JAX stock: 007900) mice were used in this study (Neonatal P1 C57BL/6 J mice was used for primary microglia cell culture only). A total of ~ 250 adult mice and ~ 30 neonatal mice were used in this study. Mice were housed in polypropylene cages and maintained at 25 °C under reverse phase 12-h light–dark cycle with ad libitum access to water and rodent chow. Animals were tagged and randomly allocated to each group before any procedures. All procedures were carried out with the approval of the Animal Care Committee of the First affiliated Hospital at Zhejiang University.

### Tibial fracture surgery

As previously reported[29], surgery was consisted of an aseptic open tibial fracture with intramedullary fixation performed under general anesthesia, including 1.5% isoflurane (RWD Life Science, Shenzhen, China) in 800 mL/min O_2_ using a rodent inhalation anesthesia apparatus (MIDTRX VIP2000, Midmark, Dayton, OH, USA). Briefly, the left hind limb was disinfected. Following the skin incision, a 28G needle (~ 0.38 mm) was inserted into the tibial intramedullary canal. Osteotomy was then performed at the junction of the middle and distal thirds of the tibia. The fixation needle remained in situ and be cut flush with the tibial cortex. After producing the fracture, skin was closed with 5–0 Vicryl sutures (Monocryl; Ethicon Inc, Somerville, NJ); thereafter, animals were allowed to recover spontaneously from anesthesia. During surgery and recovery, body temperature was maintained at 37 °C with a warming pad. The entire procedure from the induction of anesthesia to the end of surgery took less than 15 min. Tramadol (30 mg/kg) was administered intraperitoneally daily for analgesia. Control (sham-treated) mice received same anesthesia and analgesia as for tibial fracture surgery.

### Trace-fear conditioning (TFC) test

TFC test was used to assess learning and memory in mice[29]. Freezing behavior is an indicator of aversive memory which is evoked by contextual cues related to a fear-inducing stimulus–response pairing learned during training. The extent of this learning (contextual fear) was recorded in a TFC chamber (Shanghai XinRuan Information Technology Co., Ltd, Shanghai, China). Memory impairment was indicated by a decrease in freezing time.

#### Training

Before start of behavioral tests, mice were habituated to handling by the researcher for 3 days. In each day, mice were handled by the researcher for 2 min in the experimental room. On the training day, mice were placed in the TFC chamber and allowed to explore for 100 s. Mice were then exposed to an auditory cue (75–80 Db, 5 kHz, conditional stimulus) for 20 s, followed 20 s later by a 2 s foot shock (0.8 mAmp; unconditional stimulus). The tone and foot-shock pairing were repeated with an inter-trial interval of 100 s. After training, mice were allowed to remain in the testing chamber for 1 min before returning to the home cage.

#### Contextual testing

Three days after the training session, mice were placed back in the same TFC chamber without any tone or shock for 5 min. Video tracking system recorded the time spent freezing as a fraction of total time in the chamber.

### Novel object recognition test (NOR test)

The Novel Object Recognition task was used to evaluate general cognitive function. This test was based on the spontaneous tendency of rodents to spend more time exploring novel object than a familiar one. The NOR test was conducted in an open field arena with two kinds of objects different in shape and appearance, but were generally consistent in height and volume. Behavior was videotaped and recorded by Anymaze software (*Stoelting Co.*, IL, USA). Mice were habituated to handling by the researcher for 3 days before the start of behavioral tests. Animals were exposed to the arena with two identical objects placed in opposite corners for 10 min. 24 h later, mice were returned to the testing environment, where one of the sample objects was replaced by a novel object that different from familiar object in shape and texture for 5 min. The testing session was scored by an experienced researcher who was blinded to the experimental condition. Animals were scored as exploring an object when the animal spent with its head and nose oriented toward and within 2 cm of the object. Accidentally touching, sitting or standing on the object were not considered exploratory activity. The discrimination ratio was calculated as the time spent exploring the novel object divided by the total time spent with both novel and familiar objects. Higher ratio indicates better recognition performance.

### Cytokine measurement

Whole blood was collected by ventricle while mice were under deep anesthesia. Blood samples were centrifuged at 3,000 rcf/min for 15 min, serum was then collected and stored at −20 °C until assayed. Then mice were immediately perfused with saline and hippocampus tissues were then rapidly extracted and disrupted by sonication in NP-40 lysis buffer (50 mM Tris, PH 7.4, 150 nM NaCl, 1% NP-40, 0.1% Triton X-100 and 0.1% SDS) containing protease inhibitors (*Beyotime Biotech*, China). The homogenized material was centrifuged at 20,000*g* for 15 min, and the cleared supernatant was collected for analysis. Total protein levels in homogenates were determined by the BCA assay (*Beyotime Biotech*, China). IL-6 and LCN2 protein levels were measured using the corresponding Mouse ELISA kit (*R&D systems*, USA), respectively. Samples were processed according to the manufacturer’s instructions.

### MACS (magnetic activated cell sorting)

The hippocampus was dissected and stored in 1 mL cold HBSS buffer (hippocampus from 5 mice for Sham and Surgery group, respectively). The hippocampus was minced and dissociated in 8 mL of a mixture containing collagenase IV (5 mg/ml), DNase I (5 mg/ml) and 5% FBS, followed by bathing in water at 37 °C for 30 min. Cell suspension was then filtered through 70 µm cell strainer (BD Falcon, USA). Myelin debris was removed by using Myelin Removal Beads II (Miltenyi Biotec, Germany). Cell suspension was centrifuged at 300 g/min for 10 min then resuspended in 0.5% BSA. After incubating with anti-CD11b microbeads (Miltenyi Biotec, Germany) on ice for 15 min, CD11b^+^ and CD11b^−^ cells were isolated by MiniMACS separator (Miltenyi Biotec, Germany) according to the manufacturer’s instructions.

### Microglia depletion

To deplete microglia, *cx3cr1*^CreER±^ mice were crossed with *Rosa26*^iDTR±^ mice. *cx*_*3*_*cr1*^CreER±^::*R26*^iDTR±^ mice were orally gavaged with tamoxifen (0.35 mg/g body weight, dissolved in corn oil) or corn oil (for control group) once daily for 3 days, which induces the expression diphtheria toxin receptor (DTR) in *cx*_*3*_*cr1*-positive cells. As microglia are self-renewing with a slow turnover rate (months to years) as compared with that of *cx*_*3*_*cr1*-positive monocytes (< 1 week), only microglia continue to express DTR weeks after tamoxifen administration [35, 36]. Four weeks after tamoxifen treatment, mice were treated with diphtheria toxin (1 µg per mice, sigma) via intraperitoneal (ip) injection once daily for 3 days.

### rLCN2 protein and viral intracranial injection.

*rLCN2 protein intracranial injection* 12-week-old mice were anesthetized under 1.5% isoflurane, and cannulas were implanted in CA1 region bilaterally (AP: −2.5, ML: ± 2.0, DV: −1.8). The cannula assembly was secured to the skull with dental cement, and mice were allowed to recover for 3 weeks. After TFC training, mice were immediately anesthetized under 1.5% isoflurane, and mouse rLCN2 (250 µg/µL, 500 nL, dissolved in PBS buffer, R&D systems, USA) was injected bilaterally using a 10μL syringe with a 30-guage metal needle (Hamilton Instruments) at a slow rate manually. After injection, the needle was left in place for another 5 min and then withdrawn slowly. Control group received vesicle. The mice were killed after behavior test and the location of injection site was examined by microscopy.

#### Viral intracranial injection

To knockdown of LCN2, AAV viral delivery of shRNA was conducted. Constructs were generated including LCN2-AAV (pAKD-CMV-bBlobin-eGFP-H1-shRNA-LCN2, 6.25 × 10^12^ GC/mL titer, *Obio technology corp*, Shanghai, sequence listed in Additional file 3: Table S2) and Control-AAV (pAKD-CMV-bBlobin-eGFP-H1-shRNA-Control, 8.0 × 10^12^ GC/mL titer, *Obio technology corp*, Shanghai). For virus injection, 12-week-old mice were anesthetized under 1.5% isoflurane. After a small craniotomy was performed, 200 nL virus was delivered (AP: −2.5, ML: ± 2.0, DV: −1.8) using a 10 μL syringe with a 30-guage metal needle (Hamilton Instruments). The flow rate (50 nL/min) was controlled by an injection pump. After injection, the needle was left in place for another 10 min and then withdrawn slowly. Knockdown efficiency was assessed by measuring *Lcn2* mRNA and protein expression in the hippocampus 3 weeks after the injection of LCN2-AAV or Control-AAV. The location of eGFP expression (infection efficiency of virus) was also examined by fluorescent microscopy. Only mice with correct injection locations and efficient viral expression were used for further analysis.

### Cell culture (BV2 microglia cell line and mouse primary microglia)

BV2 microglia cell line (*ATCC No. CRL1834*) was purchased from the Institute of Basic Medical Sciences, Chinese Academy of Science. Cells were maintained in high glucose DMEM supplemented with 10% FBS and 1% penicillin/streptomycin under humidified 5% CO_2_ air environment at 37 ºC. Mouse primary microglia cultures were obtained from the cortex of neonatal C57BL/6 J mice (P1). In brief, mouse forebrains were incubated in 0.25% trypsin for 15 min at 37 ºC, and reaction was stopped by addition of the culture medium (DMEM with 10% FBS, 1% penicillin/streptomycin). Then the cortices were dissociated by repeated up- and down-pipetting with a 5 ml pipette. The resulting cell suspension was centrifuged at 700 rcf/min for 5 min. The pellet was triturated with culture medium, passed through a 70-μM nylon cell strainer, and then centrifuged again. Then resuspended cells were seeded in T-75 flasks in culture medium at 37 ºC with 5% CO_2_. Two weeks later, culture flasks were shaken for 1 h at 250 rpm/min on an orbital shaker (37 ºC) to harvest microglia. The medium containing detached microglia was collected and immediately centrifuged for 10 min at 1000rcf/min. Cells was resuspended with fresh culture medium and seeded (3.5 × 10^5^ cells/well on a 6-well plate). The purity of the microglial cultures was > 95% as determined by immuno-histochemistry with Iba-I antibody. Mycoplasma contamination PCR tests were performed prior to experiment.

### rLCN2 treatment in vitro

Twenty-four hours after seeding, rLCN2 protein (*R&D System*, USA) was added into culture medium and the final concentration was 500 ng/mL. Cell lysate and culture medium were collected at 6 h.

### qRT-PCR

Total RNA was extracted from hippocampus of mice or cells by Trizol followed by using RNA extraction kit (*Tiangen Biotech*, Beijing, China). Primer sequences are listed in Additional file 2: Table S1. qRT-PCR was performed on a CFX 96 real-time PCR thermocycler (*Bio-Rad Lab,* CA, USA). For amplification, SYBR Premix Ex Taq II (*Takara Bio Inc.*, Otsu, Japan) was used as instructed in the manufacturer's manual. Relative changes of mRNA expression were analyzed with the 2^–ΔΔ*C*t^ method with *gapdh* as an internal reference. These standardized data were used to calculate fold-changes in gene expression. All real-time PCR amplifications were performed in triplicates.

### Immunofluorescence histochemistry.

Mice were killed and perfused with saline then followed by 4% paraformaldehyde. Brains were removed and post fixed at 4 °C overnight. Brains were dehydrated in sucrose solution (30%) for 24 h at least. Coronal sections (35 μm thick) were cut on a cryostat and stored in cryoprotectant at −20 ºC. In brief, sections were washed in PBS buffer, followed by incubation with blocking medium (PBS buffer containing 10% donkey serum, 1% bovine albumin and 0.1% Triton X-100) for 1 h at room temperature. Then the sections were incubated with primary antibodies (IbaI, 1:500, *Novus*, #NBP2-19019; GFAP, 1:400, *CST*, #12389; NeuN, 1:400, *CST*, #24307; LCN2, 1:500, *R&D Systems*, #AF1857) in PBS containing 1% BSA at 4 ºC overnight. After washing 4 times for 5 min in PBS, sections were incubated with secondary antibody (TRITC-conjugated donkey anti-rabbit or AF488-conjugated donkey anti-goat, 1:400) for 1 h at room temperature. Sections were then washed in PBS, incubated with DAPI (1:1000, *CST*, #4083) in PBS for 10 min. Subsequently, sections were washed in PBS buffer, mounted onto microscope slides with anti-fade mounting medium (*Solarbio*, #S2100).

### Image acquisition

All images were acquired using a 10 X or 20 X objective lens on a confocal microscope (Nikon A1). Images were collected with an image matrix of 1024 × 1024 pixels.

### Sholl analysis

Images were opened by ImageJ software (USA, National Institute of Health). Z-stack was applied to collect 20-μm-thickness images (*n* = 4 mice/group, 9 image stacks per animal). Single microglia were isolated and thresholded manually [53]. Then, using Sholl analysis plugin, set the first shell with 5 μm and subsequent shells at 2-μm step size, and get intersection numbers at each Sholl radius. Ramification index is defined as the ratio of the perimeter to the area, normalized by that same ratio calculated for a circle of the same area. Specifically, *R* = (perimeter/area)/[2⁎(π/area)^1/2^]. Thus, *R* = 1 if the cell is a perfect circle, the larger R, the more ramified the cell. All analysis was carried out by an experienced researcher who was blinded to the experimental conditions.

### Statistics

Graphical and statistical analyses were conducted in GraphPad 6.0 (Prism Software Inc., CA, USA). Data are presented as means ± standard error of the mean (SEM). Tests for differences between two populations were performed using unpaired two-tail Student’s *t* test. Multiple-group comparison was performed with one-way ANOVA followed by a Bonferroni’s post hoc test. No data points were excluded from the statical analysis. Significance was set at *P* < 0.05.

## Results

### Tibial surgery leads to cognitive decline and LCN2 elevation in hippocampus

**Fig. 1:**
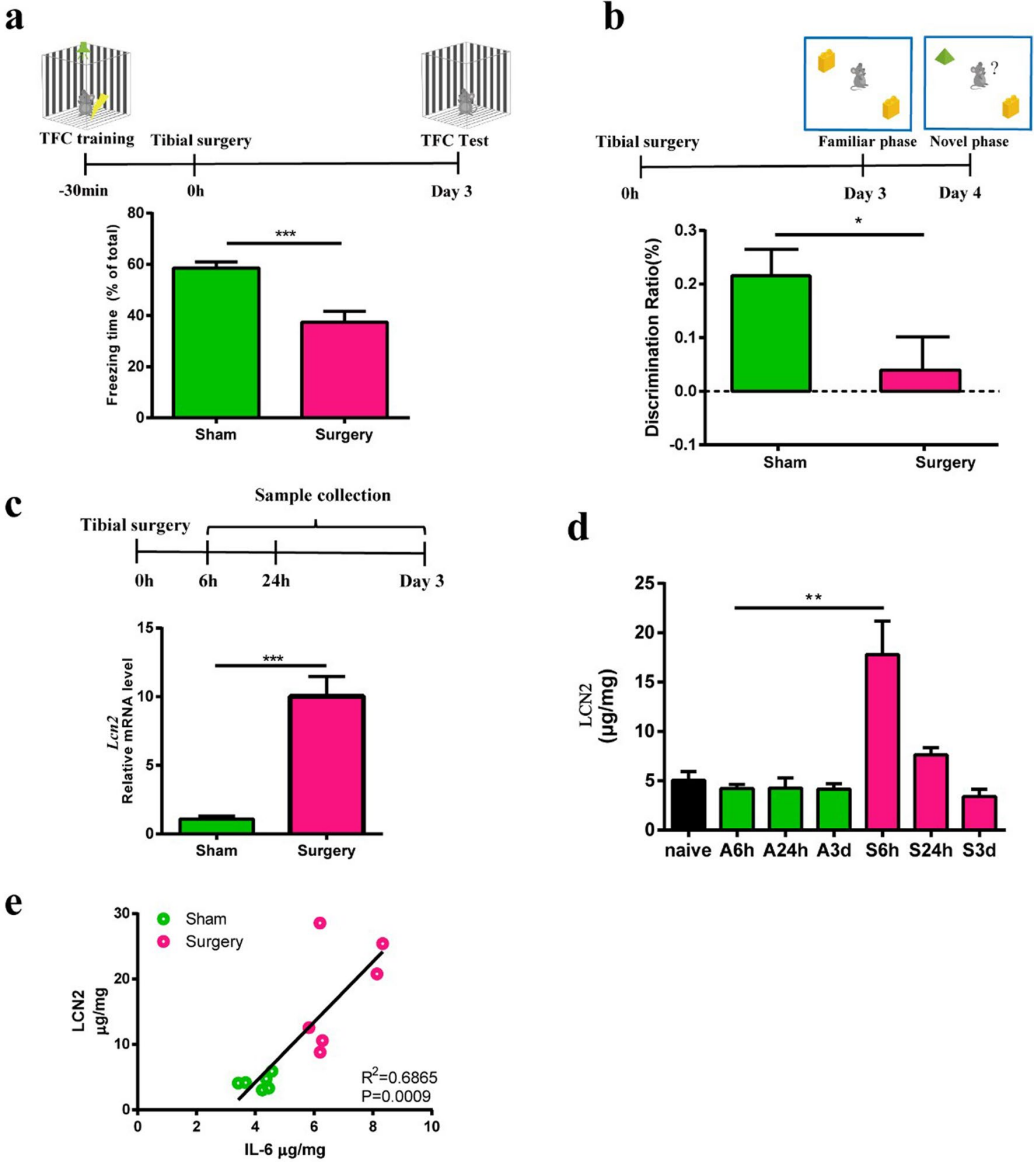
Tibial surgery leads to cognitive decline and LCN2 elevation in hippocampus. **a** Upper panel, cartoon depicting the TFC behavior paradigm. Lower panel, percent of time spent in freezing behavior (*n* = 12 mice/group). **b** Upper panel, cartoon depicting the NOR test paradigm. Lower panel, discrimination ratio during novel phase (*n* = 8 mice in Sham group, *n* = 7 mice in Surgery group). **c** Upper panel, samples were collected in another cohort at time points of 6 h, 24 h and 3 days, respectively. Lower panel, relative expression of *Lcn2* mRNA at 6 h after surgery (*n* = 5 mice/group). **d** Tissue ELISA test of LCN2 in hippocampus (*n* = 3–6 mice/group). **e** LCN2 level correlates with IL-6 level in hippocampus at 6 h. Data are represented as mean ± SEM, * P < 0.05, ** P < 0.01; *** P < 0.001

To investigate the mechanisms of PNDs, an aseptic tibial fracture surgery model ^28^ was established and cognitive functions were assessed in a trace-fear conditioning (TFC) paradigm and Novel Object Recognition (NOR) task (Fig. 1a, b). Compared with anesthesia-matched sham procedure, surgery induced memory decline in mice as evidenced by a significant reduction in freezing time (Fig. 1a). In NOR test, we found surgery had no effects on the animals’ spontaneous locomotor activity and exploration behavior, as evidenced by similar total exploration time between groups (Additional file 1: Fig. S1a). Notably, mice in Surgery group had lower discrimination ratio than Sham group, suggesting impaired recognition memory (Fig. 1b).

In our previous genome-wide RNA-seq study, we identified *Lcn2* as one of the top upregulated genes in the mouse hippocampus after surgery [28],. Here, qPCR analysis confirmed the upregulation of *Lcn2* mRNA in the hippocampus (Fig. 1c). ELISA analysis showed that the amount of LCN2 protein was elevated at 6 h after surgery (Fig. 1d), and then returned to the baseline level 3 days after surgery. Correlation analysis showed that LCN2 level was positively correlated with IL-6 level in the hippocampus at 6 h (Fig. 1e). Meanwhile, ELISA analysis showed elevation of blood IL-6 which was consistent with previously reported (Additional file 1: Fig. S1b) [29], and LCN2 was also elevated in blood (Additional file 1: Fig. S1c). In this study, we only focused on the brain cellular source of LCN2.

### LCN2 is mainly expressed in neurons

**Fig. 2:**
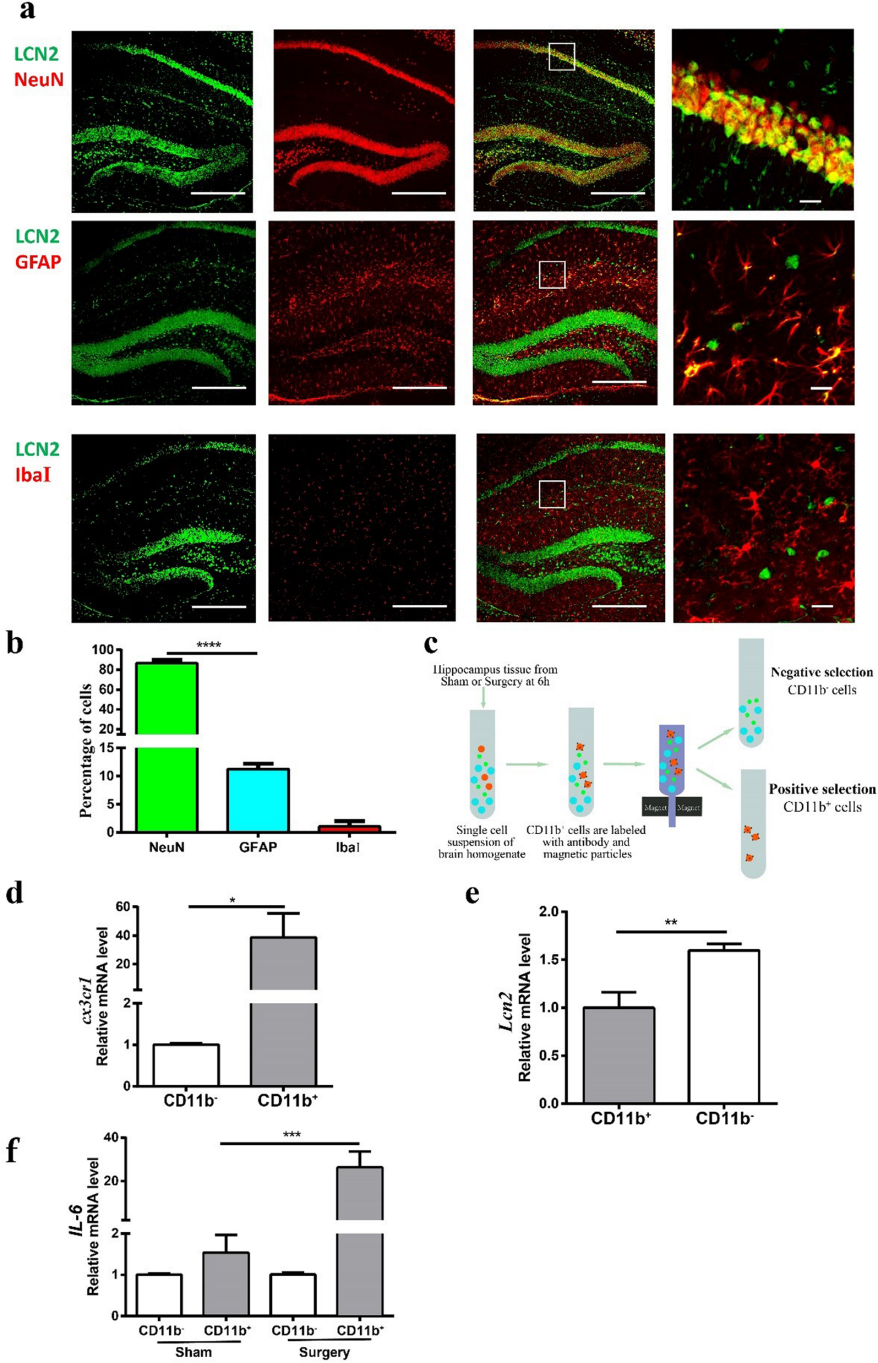
LCN2 mainly expressed on neurons. **a** Representative immunofluorescence images of LCN2 (green), GFAP (red), NeuN (red) and IbaI (red) staining in sections of the hippocampus after surgery. Long scale bar, 600 µm; short scale bar, 25 µm. **b** Percentage quantification of LCN2 positive cells (*n* = 3 mice/group). **c** Cartoon depicting MACS sorting procedures for isolating CD11b^+^ and CD11b ^−^ cells. **d** Relative expression of *cx3cr1* in CD11b ^+^ and CD11b ^−^ cells after sorting from all samples. **d** Relative expression level of *Lcn2* mRNA in CD11b ^+^ and CD11b ^−^ cells from hippocampus 6 h after surgery. **e** Relative expression level of *IL-6* mRNA in CD11b ^+^ cells. Data are represented as mean ± SEM. **** P < 0.0001

To characterize the cellular source of LCN2, mouse brains were dissected at 24 h after surgery then processed for immunofluorescence histochemistry (Fig. 2a). More than 80% of LCN2^+^ cells were co-labeled with NeuN (Fig. 2b), suggesting LCN2 was mainly expressed in neurons but not microglia or astrocytes. In a parallel experiment, CD11b^+^ microglia cells and CD11b^−^ cells were isolated by MACS (magnetic activated cell sorting) (Fig. 2c). Sorting purity was verified by *cx3cr1* expression level (Fig. 2d). Like immunofluorescence histochemistry analysis, qPCR analysis verified that *Lcn2* was expressed higher in CD11b^−^ cells than CD11b^+^ cells (Fig. 2e). Meanwhile, compared with Sham group, the expression of IL-6 was upregulated in CD11b^+^ cells in Surgery group (Fig. 2f), suggesting that microglia were switched to a proinflammatory phenotype.

### Elevation of LCN2 is independent of the presence of microglia

**Fig. 3:**
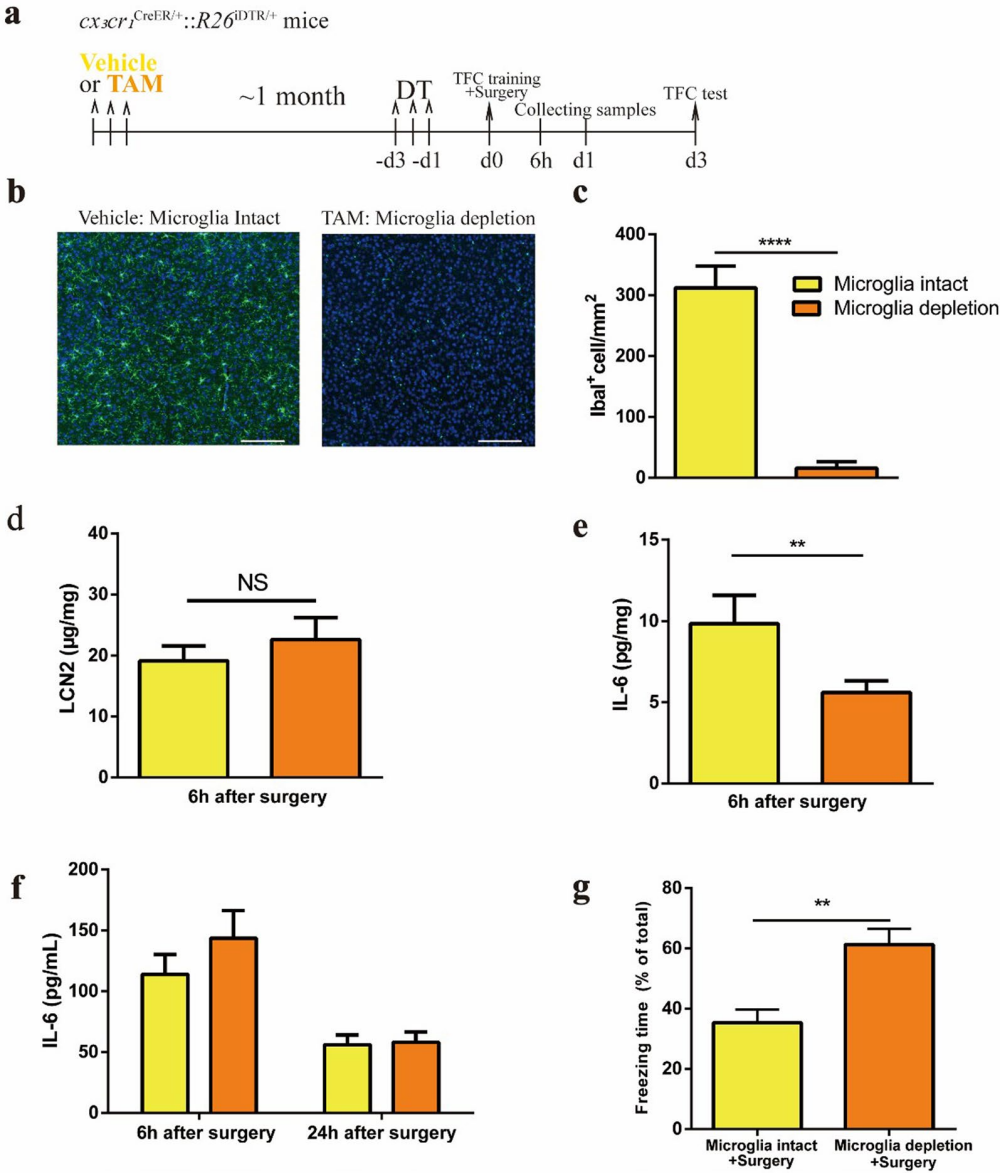
Elevation of LCN2 is independent of the presence of microglia. **a** Schematics of microglia depletion in *cx3cr1*^CreER±^:: *R26*^iDTR±^ mice. Vehicle: corn oil; TAM: tamoxifen dissolved in corn oil. **b** Representative brain sections with IbaI^+^ cells (green). Scale bars, 350 µm. **c** Quantification of the microglia density in **b** (*n* = 2 mice/group). **d** Tissue ELISA of LCN2 at 6 h after surgery (*n* = 5 mice/group). **e** Tissue ELISA of IL-6 at 6 h after surgery (*n* = 5 mice/group). **f** Serum ELISA of IL-6 at 6 h or 24 h after surgery (*n* = 5 mice/group). **g** Percent of time spent in freezing behavior (*n* = 12 mice/group). Data are represented as mean ± SEM; NS, no significance; ** P < 0.01; **** P < 0.0001

Next, we sought to determine whether the elevation of LCN2 after surgery requires microglia. CSF1R inhibitor and genetical models were commonly used approaches to effectively deplete microglia. Previous studies have shown that continuous application of CSF1R inhibitor reduces more than 30% of circulating *cx3cr1*^+^ monocytes [35] which have been reported to be critical in peripheral immune activation induced learning deficits[35]^34^. To avoid this, *cx*_*3*_*cr*_*1*_^CreER/+^:: *R26*^iDTR/+^ model was used in this study to selectively ablate microglia without affecting peripheral *cx3cr1*^+^ monocyte population ^34,35^. Induction of diphtheria toxin receptor (DTR) expression in *cx*_*3*_*cr*_*1*_^+^ cells was achieved by oral gavage of tamoxifen, leaving only microglia sensitive to DT-induced cell death after spacing a ~week period during which peripheral monocytes were renewed (Fig. 3a). After continuous 3 days of DT administration, IbaI immuno-histochemistry confirmed that more than 90% of microglia in hippocampus were depleted (Fig. 3b, c). LCN2 was still elevated after surgery after microglia depletion (Fig. 3d). In addition, the amount of IL-6 in the brain was decreased compared with the group with microglia intact (Fig. 3e), while the level of circulating IL-6 was comparable to that in microglia-intact group (Fig. 3f), suggesting activated microglia is a major contributor of neuroinflammation. Finally, in mice without microglia, cognitive function was preserved after surgery, as evidenced by increased freezing time (Fig. 3g). These data suggest that elevation of LCN2 after surgery is independent of the presence of microglia, and cognitive decline is dependent on the presence of microglia.

### LCN2 mediates cognitive dysfunction after surgery

**Fig. 4:**
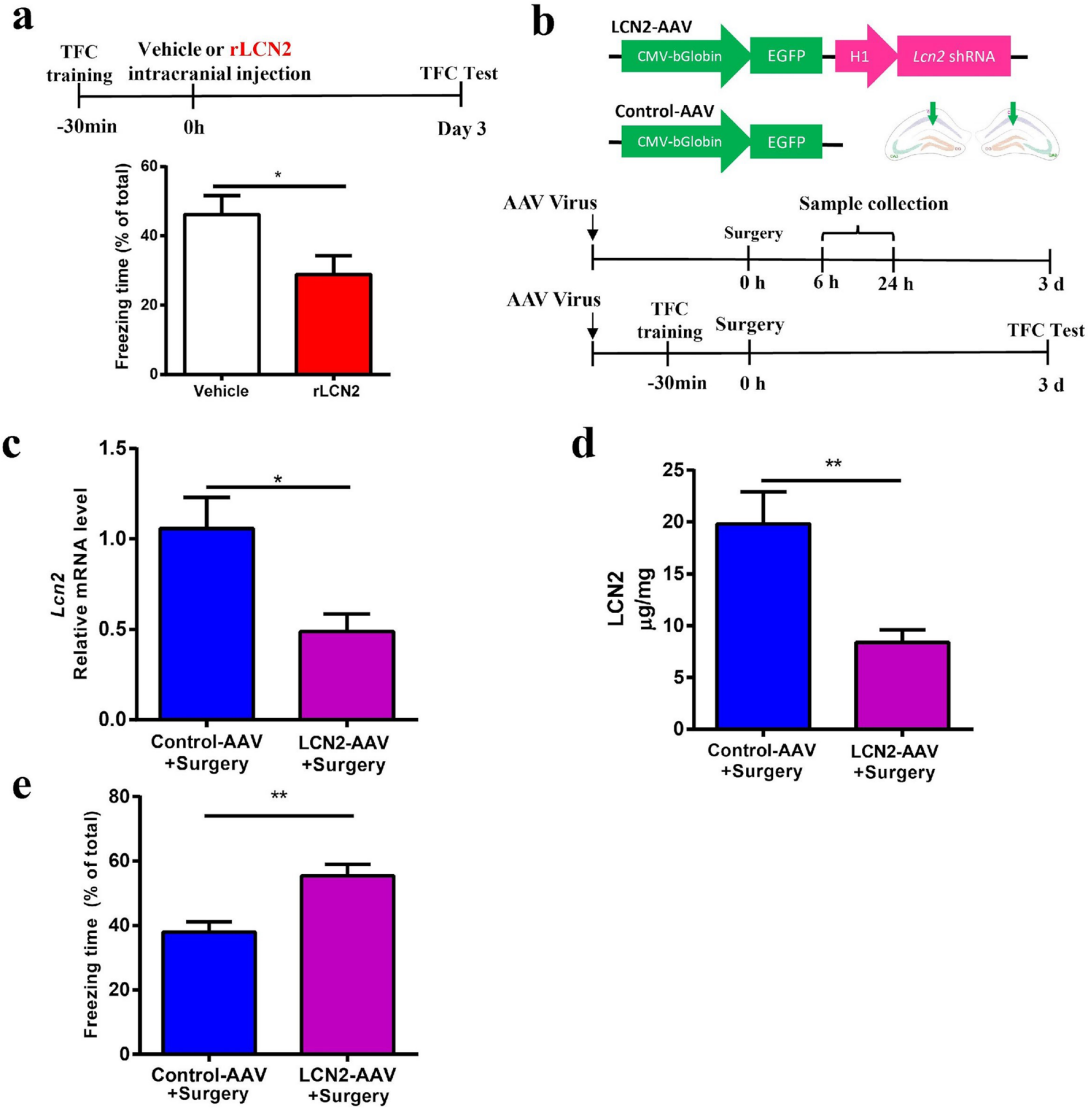
LCN2 mediates cognitive dysfunction after surgery. **a** Upper, scheme timeline for rLCN2 intracranial injection and TFC behavior test; lower, percent of time spent in freezing behavior (*n* = 8 mice in Vehicle group, *n* = 9 in rLCN2 group). Vehicle: PBS buffer. **b** Upper, constructs of the LCN2-AAV and Control-AAV; lower, scheme timeline for TFC behavior test and sample collections. **c** Relative expression of *Lcn2* mRNA at 6 h after surgery (*n* = 5 mice/ group). **d** Tissue ELISA of LCN2 level in hippocampus at 6 h after surgery (*n* = 5 mice/group). **e** Percent of time spent in freezing behavior (*n* = 12 mice/group). Data are represented as mean ± SEM, * P < 0.05; ** P < 0.01

Next, rLCN2 protein was bilaterally injected into the CA1 region of the mouse hippocampus immediately after TFC training. Memory performance was impaired as evidenced by decreased freezing time (Fig. 4a). To selectively deplete LCN2, three AAV-shRNA viral constructs were designed on different targeting *sequences;* a random sequence-scrambled shRNA-expressing AAV vector was used as a control. One of the most effective knocking down constructs was selected in this study (data not shown). To ensure effective expression, AAV viruses were bilaterally delivered into the CA1 region (LCN2-AAV: ~ 6.25 × 10^12^ GC/mL titer; Control-AAV: ~ 8.0 × 10^12^ GC/mL titer. 200 nL for each hemisphere) (Fig. 4b). Three weeks later, extensive transfection was found in neurons (> 95%), but not astrocytes nor microglia (Additional file 1: Fig. S2). qPCR and ELISA analysis verified the silencing effect of *Lcn2* (Fig. 4c, d). Freezing time was increased after knocking down of LCN2 when compared with Control-AAV group after surgery (Fig. 4e), indicating cognitive decline was ameliorated. These data suggest LCN2 mediates cognitive dysfunction after surgery.

### Knockdown of LCN2 prevents microglial activation and neuroinflammation

Recent studies suggest that microglia might play an important role in the development of PNDs [23,36], but the up- and down-stream mechanisms are far less clear. As we observed increased expression of LCN2 in neurons, we sought to examine the response of microglia to LCN2.

**Fig. 5:**
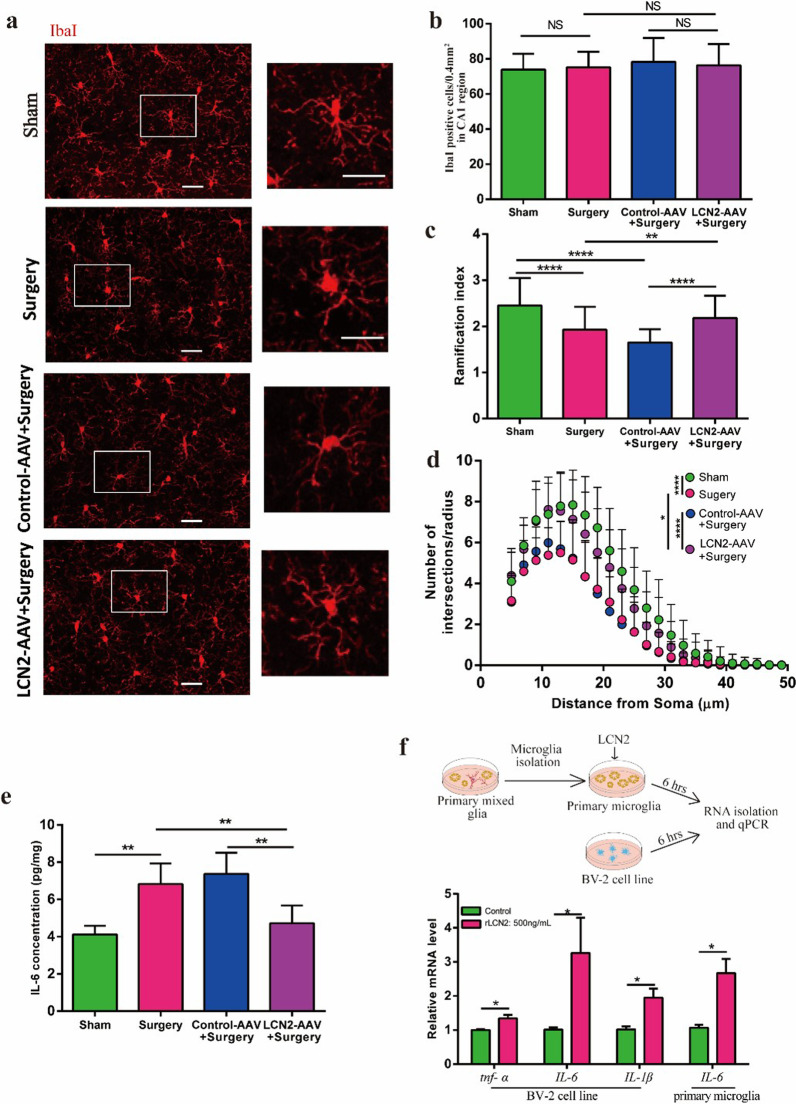
Knockdown of LCN2 prevents microglial activation and neuroinflammation. **a** Representative immunofluorescence images of hippocampal sections with IbaI^+^ cells (White). Scale bars, 150 µm. **b** Quantification of the microglia density in (A) (*n* = 4 mice/group, 2–3 slides/mice). **c** Quantification of the microglia Ramification index at 24 h after surgery (*n* = 72 cells from 4 mice/group). **d** Number of process intersections with shells at distances (in 2 µm in increments) from the soma by Sholl analysis at 24 h after surgery (*n* = 72 cells from 4 mice/group).** e** Tissue ELISA of IL-6 at 24 h after surgery (*n* = 5 mice/group). **f** Relative expression level of *tnf-α*, *IL-6* and/or *IL-1β* 6 h after application of rLCN2 (500 ng/mL) on BV-2 (*n* = 4–6/group) and primary microglia cells (*n* = 3/group). Vehicle: PBS buffer. Data represent mean ± SEM; *n* = 4/group. * P < 0.05; *** P < 0.001

Immunostaining of hippocampal slices against Iba-I was performed at 24 h after surgery (Fig. 5a). Microglial cell density remained unchanged (Fig. 5b). To quantify the complexity of microglia processes, ramification index was calculated (see Methods). Microglial ramification index in Surgery group was lower than Sham group, which was mitigated in LCN2-AAV group but not in Control-AAV group (Fig. 5c). Sholl analysis revealed that microglia in Surgery group had fewer process intersections with shells at different radii from cell soma when compared with Sham group, which was alleviated in LCN2-AAV group but not Control-AAV group (Fig. 5d). In addition, silencing LCN2 reduced the elevation of IL-6 in the mouse hippocampus after surgery (Fig. 5e). In in vitro experiments, rLCN2 were applied to BV-2 cell line and primary microglia cell culture, respectively. qPCR analysis found the expression of several proinflammatory cytokines including *tnf-α*,* IL-6*, *IL-1β* were elevated (Fig. 5f). These data suggest that silencing LCN2 prevented surgery induced switching of microglia to proinflammatory state and following neuroinflammation.

## Discussion

In this study, we provided several lines of evidence supporting the hypothesis that LCN2 serves as a signal from neuron to evoke proinflammatory microglia, contributing to surgery-induced neuroinflammation in hippocampus and memory deficits in mice. Firstly, by immuno-histology, MACS sorting and/or qPCR analysis, we showed that LCN2 was robustly expressed in hippocampal neurons and IL-6 was upregulated in microglia after surgery. Furthermore, by performing microglia depletion in vivo, we demonstrated that surgery-induced cognitive decline is dependent on the presence of microglia, while LCN2 upregulation was independent of the presence of microglia. By immune-staining and Sholl analysis, we found that after surgery, microglia were not changed in cell density in the brain but exhibit a less ramified morphology; by gain and loss of function of LCN2, we revealed that LCN2 mediates surgery-induced microglia activation and cognitive decline. Last but not least, we show that application of LCN2 elicits an proinflammatory microglia phenotype in vitro.

Although we demonstrated a robust elevation of LCN2, we did not investigate what signals trigger the expression of LCN2. Searching the regulatory upstream sequences, CCAAT-enhancer-binding site (C/EBPs), NF-kB-binding site, glucocorticoid response elements (GREs), STAT1 and STAT3 site are identified, thus numerous factors during surgery such as released DAMPs cocktail and/or surgical stress might synergically contribute to the elevation of LCN2. In a separate in vitro experiment, we found a marked elevation and secretion of LCN2 protein after application of LPS (a TLR4 activator) on a murine neuroblastoma cell line N_2_A (data not shown), suggesting the existence of the LCN2 regulation pathway in neurons.

LCN2 is initially described as a beneficial signaling involved in infectious diseases [27], the role of LCN2 in the context of non-infectious conditions is less clear.

The expression level of LCN2 is quite low in normal adult brain and elevated in many central nervous system diseases, but the source of LCN2 could come from different lines of cells such as astrocyte [37], endothelia cell [38] or neuron [39]. Our data showed that after surgery, 86% of LCN2^+^ cells were neurons and only 11.2% LCN2^+^ cells were astrocytes. Several reports revealed a detrimental role of LCN2, including impairing synaptic plasticity [40], promoting neuron death [37], or acting in a pro-inflammatory manner during ischemia [41, 42] and Alzheimer’s disease [43], and polarizing microglia toward pro-inflammatory phenotype [44, 45]. However, evidence have also shown that LCN2 may play a beneficial role in experimental autoimmune encephalomyelitis [46, 47] and sepsis models [48]. In this study, our data demonstrate a neurotoxic potential role of surgery procedure-induced neuronal LCN2, and a single dose of rLCN2 administered intracranially could lead to cognitive decline. These data are in contrast with previous reports [49], which showed that only chronic but not acute rLCN2 treatment impairs cognitive function. These discordant results can arise because of different experiment conditions such as dosage/timing of administration and behavior paradigms used. Meanwhile, limitations existed, regarding the large amount of the co-staining of LCN2 with NeuN, it raised a possibility that the other cells like astrocyte secreted or blood-derived LCN2 may also contribute to PNDs.

Microglia possess many receptors to recognize danger signals, clear cell debris and respond rapidly to changes in brain parenchyma. Accumulating evidence showed that microglia plays a central role in many neurodegenerative diseases including PNDs. Our data showed that knocking down of LCN2 could prevent microglia morphological changes and alleviates neuroinflammation after surgery. In in vitro studies, application of rLCN2 evokes proinflammatory-phenotype of microglia, but detailed mechanisms remain to be determined. Previous study showed that LCN2 promotes microglia to release inflammatory cytokines[44]. Another study showed that LCN2 could enhance priming and activation of NLRP3-inflammasome and induce mitochondria dysfunction to release reactive oxygen species in cardiac cells [50]. Meanwhile, it is also suggested that LCN2 might induce chemokine expression then activates microglia further [51]. Evaluating more cytokines and cell responses to LCN2 would provide more insights into the mechanisms underlying the effects of LCN2 on microglia.

## Conclusions

In conclusion, our previous and present studies demonstrated *Lcn2* was screened as one of the top upregulated genes in neurons after surgery. By MACS, microglia depletion, gain and loss function of LCN2, combining with in vitro experiments, we demonstrated that LCN2 might serve as a signal from neuron to microglia and contribute to the development of neuroinflammation and cognitive decline. Our findings open new avenues for research aimed at investigating pathophysiological mechanisms and developing therapeutic targets on PNDs.

## Supplementary Information

Below is the link to the electronic supplementary material.

**Additional file 1: Fig. S1.** Exploration time in NOR test and elevated cytokines after surgery. a Total exploration time in NOR test was calculated as total time spent on exploring familiar and novel objects (*n* = 8 mice in Sham group, *n* = 7 in Surgery group). b Serum ELISA of IL-6 level at 6 h and 24 h (*n* = 3–6 mice/group). c Serum ELISA of LCN2 level at 6 h and 24 h (*n* = 3–5 mice/group). Data represent mean ± SEM; * P < 0.05, ** P < 0.01, *** P < 0.001. **Fig. S2**. AAV infection in hippocampus and primary microglia cell culture. a Representative immunofluorescence images of hippocampal sections labeling with neuron (NeuN, red), astrocyte (GFAP, red), microglia (IbaI, red) and AAV infection (eGFP, green). Scale bar, 200 µm. b Representative immunofluorescence images of primary microglia culture (blue: DAPI; red: IbaI). Scale bar, 250 µm.

**Additional file 2: Table S1. **Primer sequence.

**Additional file 3: Table S2. **Targeting sequence of AAV for Knockdown of LCN2.

## Data Availability

All data used in this study are available upon reasonable request.

## Abbreviations

PNDs: Perioperative neurocognitive disorders
LCN2: Lipocalin-2
GFAP: Glial fibrillary acidic protein
NeuN: Neuronal nuclear protein
CreER: Cre recombinase-estrogen receptor fusion protein
DTR: Diphtheria toxin receptor
DT: Diphtheria toxin
shRNA: Short hairpin RNA
DMEM: Dulbecco's modified Eagle medium
BCA: Bicinchoninic acid
HBSS: Hanks' balanced salt solution
LPS: Lipopolysaccharides
