# Supplementary figures and images for: Role of lipocalin-2 in surgery-induced cognitive decline in mice: a signal from neuron to microglia
Source: J Neuroinflammation. 2022 Apr 12;19:92. doi: 10.1186/s12974-022-02455-5 (PMC9006597; doi:10.1186/s12974-022-02455-5)

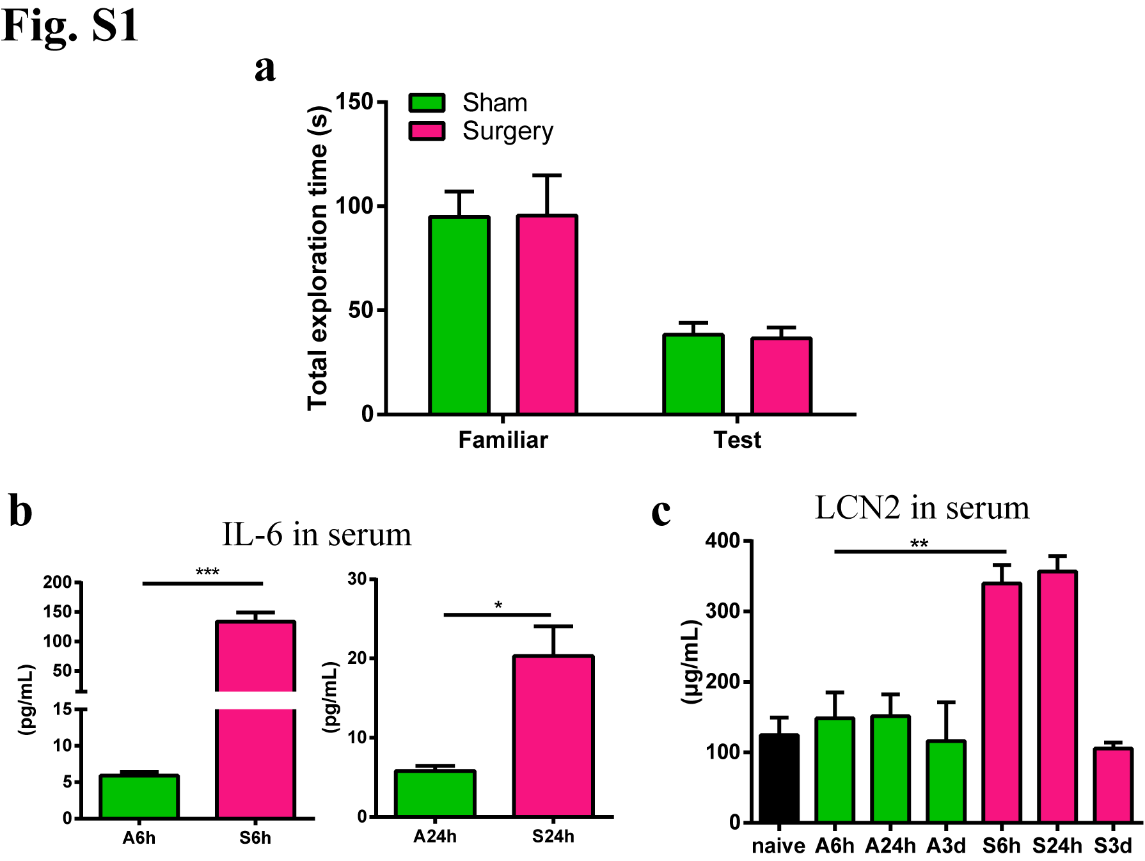
**Figure S1**

**Figure S**
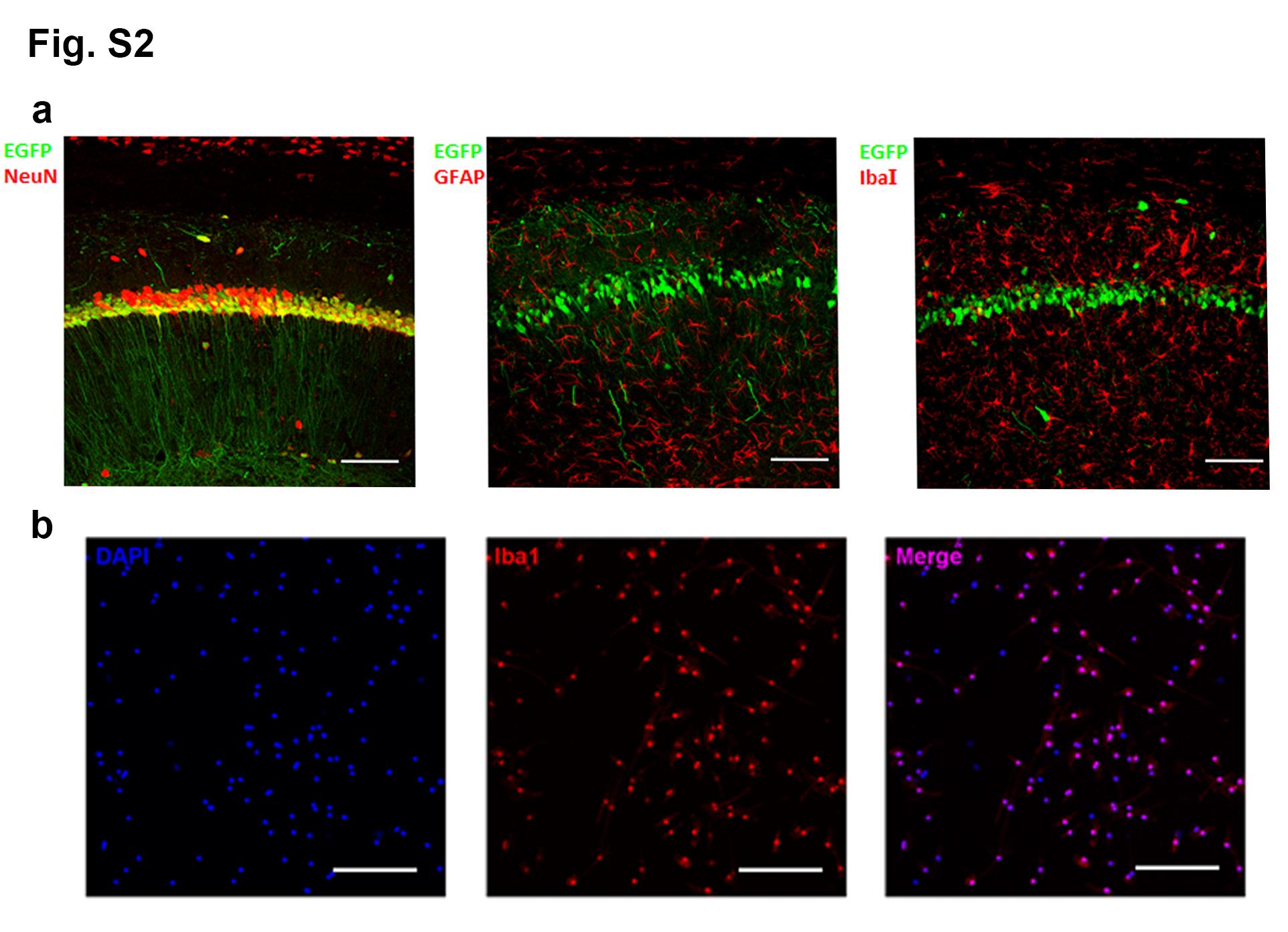
**2**

Supplement: Supplementary file 1 — Additional file 1: Fig. S1. Exploration time in NOR test and elevated cytokines after surgery. a Total exploration time in NOR test was calculated as total time spent on exploring familiar and novel objects (n = 8 mice in Sham group, n = 7 in Surgery group). b Serum ELISA of IL-6 level at 6 h and 24 h (n = 3–6 mice/group). c Serum ELISA of LCN2 level at 6 h and 24 h (n = 3–5 mice/group). Data represent mean ± SEM; * P < 0.05, ** P < 0.01, *** P < 0.001. Fig. S2. AAV infection in hippocampus and primary microglia cell culture. a Representative immunofluorescence images of hippocampal sections labeling with neuron (NeuN, red), astrocyte (GFAP, red), microglia (IbaI, red) and AAV infection (eGFP, green). Scale bar, 200 µm. b Representative immunofluorescence images of primary microglia culture (blue: DAPI; red: IbaI). Scale bar, 250 µm. [file 12974_2022_2455_MOESM1_ESM.docx]
