# Supplementary material for: Role of lipocalin-2 in surgery-induced cognitive decline in mice: a signal from neuron to microglia
Source: J Neuroinflammation. 2022 Apr 12;19:92. doi: 10.1186/s12974-022-02455-5 (PMC9006597; doi:10.1186/s12974-022-02455-5)
Supplement: Supplementary file 2 — Additional file 2: Table S1. Primer sequence. [file 12974_2022_2455_MOESM2_ESM.docx]

| Primer name | 5'->3' | gene | amplicon size(bp) |
| --- | --- | --- | --- |
| PRXXW001-TNFα-F | CTGAACTTCGGGGTGATCGG | *tnf-α* | 122 |
| PRXXW002-TNFα-R | GGCTTGTCACTCGAATTTTGAGA |  |  |
| PRXXW003-IL-1β-F | GAAATGCCACCTTTTGACAGTG | *IL-1β* | 116 |
| PRXXW004-IL-1β-R | TGGATGCTCTCATCAGGACAG |  |  |
| PRXXW005-IL-6-F | TAGTCCTTCCTACCCCAATTTCC | *IL-6* | 76 |
| PRXXW006-IL-6-R | TTGGTCCTTAGCCACTCCTTC |  |  |
| PRXXW007-GAPDH-F | AGGTCGGTGTGAACGGATTTG | *gapdh* | 123 |
| PRXXW008-GAPDH-R | TGTAGACCATGTAGTTGAGGTCA |  |  |
| PRXXW009-ccl2-F | TTAAAAACCTGGATCGGAACCAA | *ccl-2* | 121 |
| PRXXW010-ccl2-R | GCATTAGCTTCAGATTTACGGGT |  |  |
| PRXW031-Lcn2-F | GCAGGTGGTACGTTGTGGG | *Lcn2* | 95 |
| PRXW032-Lcn2-R | CTCTTGTAGCTCATAGATGGTGC |  |  |
| PRXW067-CX3CR1-F | GAGTATGACGATTCTGCTGAGG | *CX3CR1* | 102 |
| PRXW068-CX3CR1-R | CAGACCGAACGTGAAGACGAG |  |  |

**Table S1. Primer sequence.**
