# Supplementary material for: Role of lipocalin-2 in surgery-induced cognitive decline in mice: a signal from neuron to microglia
Source: J Neuroinflammation. 2022 Apr 12;19:92. doi: 10.1186/s12974-022-02455-5 (PMC9006597; doi:10.1186/s12974-022-02455-5)
Supplement: Supplementary file 3 — Additional file 3: Table S2. Targeting sequence of AAV for Knockdown of LCN2. [file 12974_2022_2455_MOESM3_ESM.docx]

| Name | Target sequence |
| --- | --- |
| pAKD-CMV-bBlobin-eGFP-H1-shRNA-NC | TTCTCCGAACGTGTCACGT |
| pAKD-CMV-bBlobin-eGFP-H1-shRNA-LCN2 | CCAGTTCACTCTGGGAAAT |

**Table S2. Targeting sequence of AAV for Knockdown of LCN2**
